# Effects of nutritional interventions on competitive performance and dose‒response relationships among esports players of different skill levels: a systematic review and three-level meta-analysis

**DOI:** 10.1080/15502783.2026.2663363

**Published:** 2026-04-24

**Authors:** Jiayi Yao, Junhao He, Enliang Hu

**Affiliations:** a School of Physical Education, China University of Mining and Technology, Xuzhou, China; b School of Physical Education and Training, Capital University of Physical Education and Sports, Beijing, China; c College of Aviation and Sports, Civil Aviation Flight University of China, Deyang, China

**Keywords:** Esports, nutritional supplements, cognitive function, competitive performance, meta-analysis, three-level model

## Abstract

**Background and objectives:**

Esports place exceptional demands on neuropsychological capacities; however, a systematic and quantitative synthesis of the specific efficacy of nutritional interventions across various tasks and competitive populations remains absent. This study aims to evaluate the impact of nutritional supplementation on cognitive function and competitive performance in esports players and to explore key moderating variables.

**Methods:**

Electronic databases, including Web of Science, PubMed, and Scopus, were searched (up to January 25, 2026). Thirteen randomized controlled trials (RCTs) met the inclusion criteria. A three-level random-effects model was employed to calculate the pooled effect size (Hedges' g), with sources of heterogeneity explored through subgroup analyses and meta-regression.

**Results:**

Nutritional interventions significantly enhanced esports-related performance (g = 0.92), with the most pronounced benefits observed in processing speed (g = 1.18) and executive function (g = 1.06). Subgroup analyses revealed significant gains in both professional (g = 0.97) and amateur groups (g = 0.92), while the improvement in first-person shooter (FPS) games (g = 0.96) appeared to surpass that in multiplayer online battle athlete (MOBA) games. Meta-regression indicated no significant linear correlation between caffeine dosage and effect size. GRADE assessment categorized the quality of evidence mostly as “low” to “moderate.”

**Conclusions:**

Nutritional interventions demonstrate significant potential for optimizing multidimensional cognitive performance in esports players, particularly in enhancing fundamental reaction speed and high-level decision-making processes. While the findings exhibit a degree of robustness, their practical application should be cautiously evaluated within specific competitive contexts due to current limitations in evidence certainty and significant interstudy heterogeneity. Future research characterized by gender balance and mechanistic orientation with long-term intervention protocols is warranted to elevate the level of clinical evidence.

## 1. Introduction

Esports (electronic sports) places rigorous demands on players' executive functions and attentional control; elite competitors are frequently required to maintain instantaneous decision-making precision under extremely high actions per minute (APM) [1,2]. Sustained and high-intensity cognitive loads are prone to inducing central fatigue, which results in a marginal decrease in reaction time and subsequent impairment of competitive performance [3,4]. Although specific technical and tactical training in esports can optimize strategic execution [5], seeking safe and effective nutritional interventions to counteract cognitive fatigue and optimize neural efficiency has become an urgent requirement for enhancing esports performance [6].

To address these demands, dietary supplements have emerged as a pivotal strategy for optimizing competitive status [7,8]. Existing research primarily focuses on two categories of interventions: one comprises stimulant supplements represented by caffeine, which aim to acutely shorten reaction times and enhance alertness by antagonizing adenosine receptors [9,10]; the other includes nootropic ingredients such as L-theanine and inositol-stabilized arginine silicate [11,12], which emphasize maintaining the stability of executive functions in complex decision-making scenarios [13]. However, current eligibility criteria for subjects in empirical studies exhibit significant heterogeneity, encompassing both professional elites and amateur players. Due to the lack of systematic quantitative integration, it remains difficult to ascertain whether interventions effective for amateur groups possess equivalent efficacy among professional players, and how the competitive level, acting as a critical moderating variable, influences the performance gains of supplementation.

Despite preliminary evidence suggesting the positive role of nutritional interventions in esports, the evidence base in this field remains highly fragmented [14], and existing reviews mostly focus on narrative summaries, lacking quantitative research across different competitive levels [15]. While significant differences exist between esports experts and amateur players in terms of baseline cognitive specialization, empirical evidence is currently lacking as to whether this individual heterogeneity, shaped by long-term competitive experience, leads to differentiated responses to nutritional interventions among different groups [16,17]. Furthermore, the heterogeneity of outcome measures, ranging from basic laboratory tasks to complex esports gameplay, obscures the true contribution of supplements to specific cognitive domains and weakens the generalizability of conclusions [18].

Based on this, the present study employs a three-level random-effects model to quantitatively integrate evidence from randomized controlled trials involving esports players of different levels, while addressing the statistical bias induced by nested effect sizes. The focus of this research lies in defining the comprehensive contribution of nutritional interventions to response speed and operational precision, and verifying whether competitive level acts as a key moderating variable affecting supplement efficacy. Through the scientific integration of cross-level evidence, this study aims to provide precise nutritional recommendations for esports participants at various performance levels.

## 2. Materials and methods

The protocol for this study was registered with PROSPERO (Registration Number: CRD420261296935). This registration explicitly defined the research objectives, inclusion and exclusion criteria, intervention and control measures, and outcome indicators. The study was conducted in strict adherence to the preregistered protocol without significant deviation and was implemented and reported according to the Preferred Reporting Items for Systematic Reviews and Meta-Analyzes (PRISMA 2020) checklist [19].

### 2.1. Search strategy

A systematic search was conducted across the PubMed, Embase, Web of Science, Scopus, Cochrane Library, and CINAHL databases, covering the period from their inception to January 25, 2026. The search strategy combined medical subject headings (MeSH) and free-text terms, focusing on core dimensions including participants, interventions, and study types. Participants included esports players, elite or amateur gamers, and habitual video game players. Interventions involved nutritional strategies such as caffeine, r-aminobutyric acid (GABA), herbal extracts, and multi-ingredient energy drinks. Study types were strictly limited to randomized controlled trials (RCTs). Boolean operators “OR” were used to expand terms within each dimension, while “AND” was used to cross-reference between dimensions. Additionally, supplementary searches were performed by tracing the reference lists of included studies and relevant reviews. Detailed search strategies for each database are provided in Supplementary Text 1.

### 2.2. Inclusion and exclusion criteria

Inclusion and exclusion criteria were established based on the PICOS principle. Inclusion criteria specified that subjects must be professional players, semi-professional players, or active players with long-term gaming habits. Interventions must involve the ingestion of dietary supplements or functional foods, including caffeine, nootropic amino acids, herbal extracts, and complex nutritional formulas. The control group must receive a placebo matched in appearance and taste. Outcome measures were required to include standardized assessment data regarding esports performance or cognitive processing. The study design was strictly limited to randomized controlled trials. Exclusion criteria included nonrandomized controlled designs or single-group pretest and posttest studies; clinical samples with neurodevelopmental disorders or those taking psychotropic medications; interventions involving only nonnutritional external stimuli or psychological training; studies with severely missing outcome data or data presentation formats that prevented the extraction of means and standard deviations for quantitative synthesis; and duplicate publications.

### 2.3. Data collection

Data extraction was performed independently by two researchers, and any discrepancies were resolved through third-party arbitration. The extracted information encompassed three primary dimensions: basic study characteristics, intervention protocols, and outcome measures. Basic characteristics included the author, year of publication, sample size, age, sex ratio, and competitive level, which categorized participants as elite, active, or amateur players. Intervention protocols comprised supplement ingredients, detailed dosages such as mg/kg or fixed amounts, frequency of administration, and game genres including FPS, MOBA, or specific cognitive tasks. Outcome measures involved the extraction of means and standard deviations (SD) for each cognitive dimension and competitive performance metric. For studies reporting multiple outcome indicators, this research extracted all eligible effect sizes to construct a three-level meta-analysis model. In cases where standard deviations for change values were missing, estimations were conducted by setting a correlation coefficient according to the Cochrane Handbook. If the required data remained unavailable after these steps, attempts were made to contact the original authors, or the study was excluded in accordance with the established screening protocol [20,21]

### 2.4. Risk of bias and certainty of evidence

The risk of bias for the included studies was independently assessed by two researchers using the Cochrane Risk of Bias tool (RoB 2.0) [22]. The assessment covered five domains: the randomization process, deviations from intended interventions, missing outcome data, measurement of the outcome, and selection of the reported results. The risk level for each domain was categorized as “low risk,” “some concerns,” or “high risk.” Discrepancies in the assessment were resolved through collective discussion or adjudication by a third senior researcher. Furthermore, the GRADE system was employed to evaluate the certainty of evidence for core outcome indicators [23]. This evaluation integrated five dimensions: risk of bias, inconsistency, indirectness, imprecision, and publication bias. The quality of evidence was ultimately classified into four levels, namely high, moderate, low, and very low, aiming to provide graded evidentiary support for the efficacy of nutritional supplements in improving the competitive performance and cognitive function of esports players.

### 2.5. Data analysis

Statistical analyzes were performed using the metafor library within R software version 4.3.1. Given that most included studies reported multiple correlated outcome indicators for the same group of subjects, a three-level random-effects model was constructed. This approach effectively handles the nesting relationship among effect sizes and controls for intra-study autocorrelation. The model decomposes the total variance into three independent levels: Level 1 represents within-subject variation (sampling error); Level 2 represents within-study variation (differences between various outcome indicators within the same study); and Level 3 represents between-study variation (differences across different studies). Likelihood ratio tests (LRT) were utilized to compare the goodness of fit between the three-level model and the traditional two-level model, while the Akaike information criterion (AIC) was used to evaluate model explanatory power [24,25]. Effect sizes were expressed as Hedges' g with 95% confidence intervals (CI), including a correction for small-sample bias based on the sample size of each study. To ensure the consistency of effect size direction, certain original outcome indicators underwent reverse processing. Specifically, for indicators where a numerical increase represents a decline in performance, such as reaction time, number of errors, or time to complete a task, the effect sizes were assigned negative values. Consequently, a positive value across all outcome indicators consistently represents an improvement in cognitive function or competitive performance. Heterogeneity assessment was achieved by calculating I^2^ values for each level, reflecting the contribution of variance at different tiers to the total variance [26]. For outcome indicators with significant heterogeneity, subgroup analyzes and meta-regression were further conducted. Subgroup analyzes primarily focused on competitive level (professional or elite versus amateur) [16], game genre (FPS versus MOBA) [5], and cognitive dimensions [27]. Meta-regression analysis was employed to explore the linear relationship between supplement dosage, such as milligrams of caffeine, and effect sizes. The assessment of publication bias combined visual observation with quantitative testing. First, multicolor annotated funnel plots were plotted to visually judge symmetry, followed by the application of Egger's regression test modified for three-level structures for quantitative identification. All statistical tests were two-sided, and a value of *P* < 0.05 was considered statistically significant.

## 3. Results

### 3.1. Study selection

**Figure 1.**
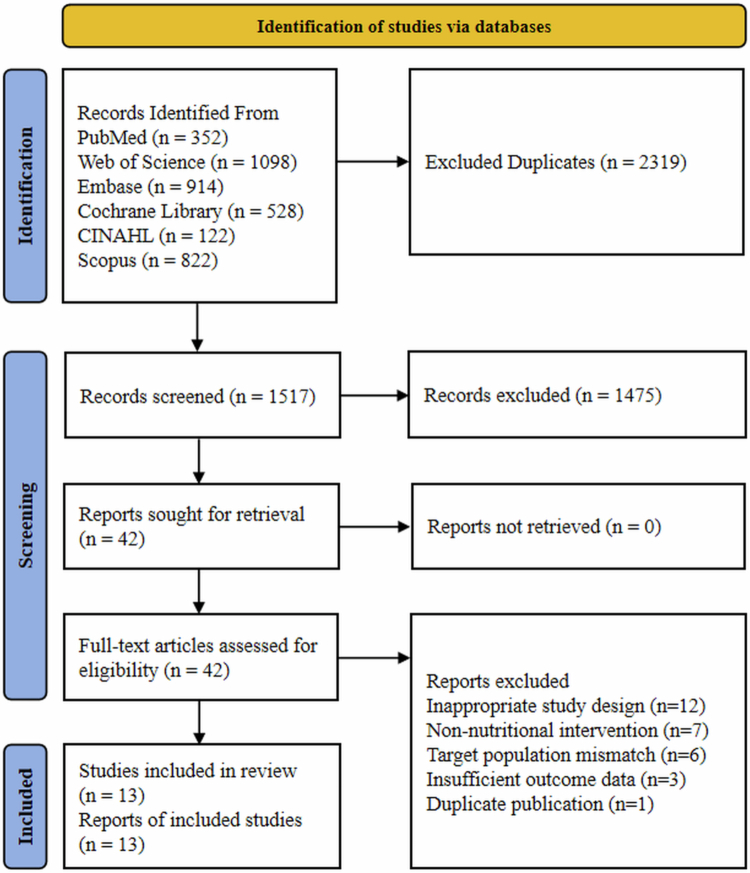
Preferred reporting items for systematic reviews and meta-analysis (PRISMA) study flow diagram.

A systematic search across PubMed, Scopus, Web of Science, Embase, Cochrane Library, and CINAHL initially identified 3,836 records. After removing duplicates and screening titles and abstracts, 42 potentially relevant studies underwent full-text review. Based on the eligibility criteria, 29 studies were excluded for the following reasons: mismatched study design (*n* = 12), nonnutritional intervention (*n* = 7), ineligible population (*n* = 6), incomplete outcome data (*n* = 3), and duplicate publication (*n* = 1). Ultimately, 13 randomized controlled trials (RCTs) were included in the meta-analysis (Figure 1).

### 3.2. Study characteristics

A total of 13 original studies [7, 12, 28–38] published between 2019 and 2025 were included in this research (Table 1) [12,37]. Regarding participant characteristics, the analysis encompassed 743 subjects, with sample sizes in individual studies ranging from 18 to 122 [28,37]. The subjects were generally young, with mean ages between 20.4 and 24.5 years [35,37]. The gender distribution reflected prominent characteristics of the esports industry, showing a very high proportion of male participants. Specifically, 10 studies utilized all-male samples, while only 3 studies included female subjects, with the female proportion in those instances being below 20% [30,35,38]. The competitive levels of the subjects covered the full spectrum from amateur and active players to professional or elite competitors. Notably, studies such as Wu et al. (2024) conducted in-depth investigations into the in-game performance of elite players, which significantly enhanced the specificity and practical application value of the results [29]. In terms of experimental design and intervention protocols, all included studies employed a randomized controlled trial (RCT) design. The active ingredients in the intervention groups were diverse. Caffeine was the most extensively studied component, including anhydrous caffeine [36], AmaTea guayusa extract [30], and multiingredient energy drinks [38]. Typical dosages were 2 to 3 mg/kg or a fixed dose of 200 mg [30,33,37]. Additionally, some studies explored the effects of r-aminobutyric acid (GABA) [12], Bacopa monnieri [31], noolvl (inositol-stabilized arginine silicate) [28], and various complex nootropic formulations such as the CDT combination or Stadice [38]. Control groups uniformly utilized placebos such as microcrystalline cellulose or maltodextrin.

**Table 1.** Basic information of the included studies.

First author and year	Mean age (Mean ± SD)	Sample size and sex ratio (M/F)	Skill level	Game genre	Intervention (ingredients and dosage)	Placebo	Outcome measure categories
Bloomer et al., 2022 [30]	23.5 ± 4.2	*N* = 98 (84 M/14F); 85.7% M/14.3% F	Active	FPS (training simulation)	200 mg AmaTea: Ilex guayusa extract (containing 40 mg caffeine and 160 mg polyphenols)	Microcrystalline cellulose capsules	Stroop color-word interference (time), reaction time (CPT)
Evans et al., 2023 [7]	24.42 ± 4.52	*N* = 49 (100% M)	Amateur	FPS (Aim Lab shooting task)	CDT combination: 200 mg caffeine, 10 mg TeaCrine, and 50 mg Dynamine	Maltodextrin capsules	Aiming task accuracy, simulated game score, decision making speed
Hara et al., 2025 [12]	22.4 ± 3.1	*N* = 60 (100% M)	Active	FPS (Apex Legends simulation)	100 mg *γ*-Aminobutyric Acid (GABA): Fermentation-derived active ingredient	Dextrin capsules	Choice reaction time, working memory capacity, attention shift speed
Jeyakodi et al., 2024 [35]	24.5 ± 3.6	*N* = 60 (48 M/12F); 80% M/20% F	Active	Integrated Esports (Multiple mainstream titles)	300 mg Stadice: Mangifera indica leaf extract (containing ≥ 60% mangiferin)	Microcrystalline cellulose capsules	Visual search time, target locking accuracy, sustained attention score
Leonard et al., 2023 [28]	24.2 ± 4.1	*N* = 122 (100% M)	Amateur or Active	MOBA (League of Legends)	1.6 g nooLVL: 1,500 mg bonded Arginine Silicate Inositol (ASI) and 100 mg inositol	1.6 g Maltodextrin capsules	Trail making test (Part B time), cognitive flexibility index
Rogers et al., 2024 [36]	21.9 ± 2.5	*N* = 40 (100% M)	Active	FPS (Aim Lab training)	3 mg/kg Caffeine Anhydrous: Precise weight-based dosage	Cellulose capsules	Kill/death ratio (KDA), simulated esports performance score
Sainz et al., 2020 [33]	21.5 ± 2.7	*N* = 30 (100% M)	Professional	FPS (CS:GO)	3 mg/kg Caffeine: Weight-based dosage in capsule form	Cellulose capsules	Simple reaction time, stop-signal reaction time (SSRT), shooting accuracy
Schwager et al., 2024 [38]	22.8 ± 3.2	*N* = 48 (42 M/6F); 87.5% M/12.5% F	Active	Cognitive competitive tasks (Reaction and Attention)	Multi-ingredient energy drink: 200 mg caffeine, 1,000 mg taurine, and B-vitamins	Non-active control beverage	Flanker task (inhibition speed), go/no-go accuracy
Seesurn et al., 2025 [31]	21.6 ± 2.8	*N* = 48 (100% M)	Active	Multi-genre (Including MOBA and FPS)	Complex nootropic formulation: 300 mg Bacopa monnieri, 200 mg L-theanine, and 100 mg caffeine	Flavor-matched maltodextrin solution	Visual processing speed, mental rotation speed, task switching latency
Sowinski et al., 2021 [32]	23.1 ± 3.9	*N* = 52 (100% M)	Active	Simulated competitive tasks (Laboratory-based)	1.6 g nooLVL: 1.5 g ASI and 100 mg free inositol	1.6 g Maltodextrin capsules	PVT reaction time, digit symbol substitution (speed)
Tartar et al., 2021 [34]	24.1 ± 4.3	*N* = 100 (100% M)	Active	Integrated Esports (PC-based testing)	CDT combination: 125 mg caffeine, 75 mg Dynamine, and 50 mg TeaCrine	Cellulose capsules	*N*-back task accuracy, error rate (inhibition), P300 latency
Thomas et al., 2019 [37]	20.4 ± 1.8	*N* = 18 (100% M)	Elite or Professional	MOBA (League of Legends)	2 mg/kg Caffeine: Administered via Red Bull® energy drink	Caffeine-free and sugar-free placebo beverage	Executive control index, reaction time variability
Wu et al., 2024 [29]	20.8 ± 1.2	*N* = 18 (100% M)	Elite	FPS (Shooting simulation and Stroop)	3 mg/kg Caffeine: Single dose capsule 60 minutes before testing	Cellulose capsules	Visual search response time, hit accuracy, kill ratio (simulation)

Notes: M/F: Male/Female; N: Sample size; Mean ± SD: Mean ± Standard Deviation; ASI: Arginine Silicate Inositol; CDT: A combination formulation of Caffeine, Dynamine, and TeaCrine; GABA: r-Aminobutyric Acid; mg/kg: Milligrams per kilogram of body weight; FPS: First-person shooter; MOBA: Multiplayer Online Battle Arena; CS:GO: Counter-Strike: Global Offensive; KDA: Kill/Death/Assist ratio; PVT: Psychomotor Vigilance Test; SSRT: Stop-signal reaction time.

Furthermore, this study categorized outcome indicators into five core sub-dimensions to systematically evaluate the transformation pathway of nutritional supplements from fundamental cognitive processing to high-level competitive performance. The classification criteria are as follows: processing speed encompasses simple and complex reaction times to visual stimuli; executive function focuses on assessing cognitive flexibility, working memory updating, and decision-making scheduling abilities; attention and Inhibition examines the capacity of subjects to maintain focus and inhibit impulsive responses in distracting environments, such as through Stroop effect control; accuracy targets precision metrics such as shooting hit rates or clicking accuracy; and game performance integrates comprehensive quality measures including kill ratio, game scores, and the completion of simulated competitive tasks. This systematic classification provides robust evidence for comprehensively evaluating the cross-dimensional impact of supplements on esports performance.

### 3.3. Risk of bias

**Figure 2.**
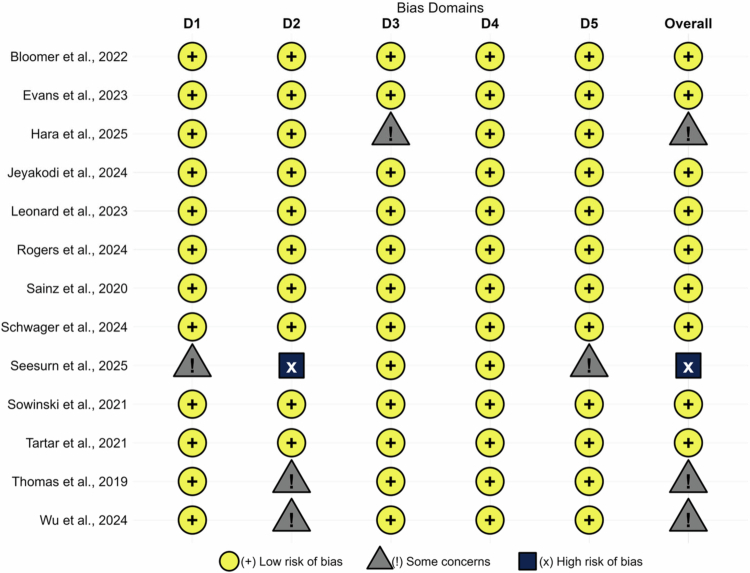
Risk of bias summary: review of the authors' judgments about each risk of bias item for each included study.

**Figure 3.**
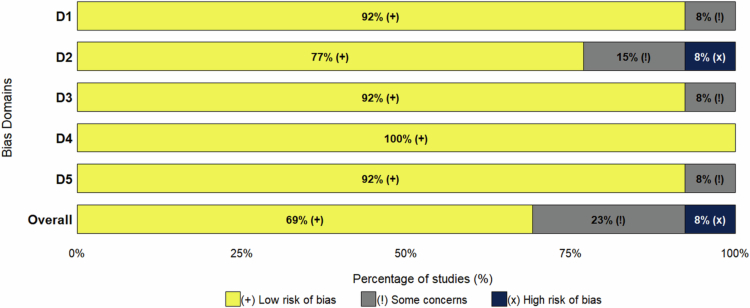
Risk of bias graph: review authors' judgments about each risk of bias item, presented as percentage of included studies.

The quality of the 13 included studies was evaluated across five domains using the RoB 2.0 tool: randomization process, deviations from intended interventions, missing outcome data, measurement of the outcome, and selection of the reported results. The detailed results are presented in Figures 2 and 3. Overall, 9 studies were categorized as “low risk” (e.g. Rogers et al., 2024; Schwager et al., 2024) [7, 28, 30, 32–36, 38]. These studies ensured the reliability of the evidence through rigorous double-blind designs and allocation concealment protocols. Three studies were assessed as having “some concerns,” primarily due to the implementation of single-blind designs (e.g. Wu et al., 2024) [29] or a lack of detailed description regarding randomization procedures in the context of small sample sizes (e.g. Thomas et al., 2019; Hara et al., 2025) [12,37]. Only one study (Seesurn et al., 2025) [31] was rated as “high risk” because of insufficient methodological transparency and the accumulation of risks across multiple domains. Taken together, the majority of the included studies are of high quality, providing a robust evidentiary foundation for this meta-analysis.

### 3.4. Meta-analysis results

**Figure 4.**
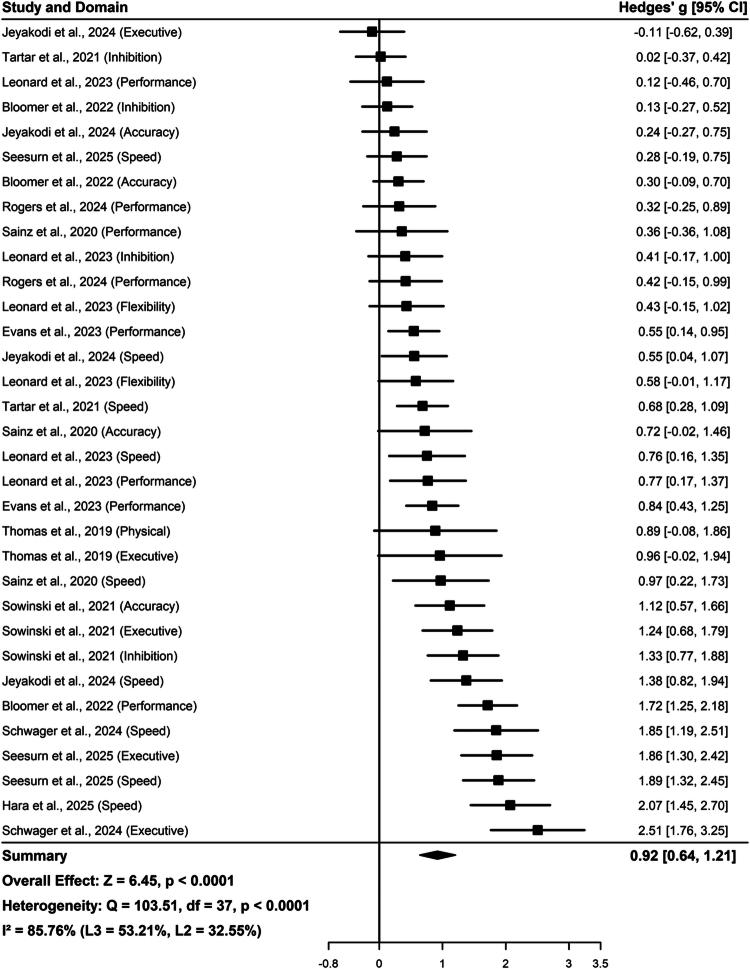
Forest plot of the three-level random-effects model.

In this study, the goodness of fit of the models was first evaluated using the likelihood ratio test (LRT). Results indicated that the improvement of the three-level model compared to the traditional two-level model did not reach statistical significance (P_LRT_ = 0.091). However, further comparison revealed that the Akaike information criterion value for the three-level model (AIC = 38.52) was lower than that of the two-level model (AIC = 39.38), suggesting that the three-level architecture may possess superior fitting indices and explanatory power. Considering that this study included several reports with multiple outcome indicators, and that the heterogeneity decomposition showed intra-study variation (level 2) contributed 32.55% of the total variance, the three-level random-effects model was ultimately selected as the primary analysis model to remain conservative and effectively control for intra-study autocorrelation. After incorporating 38 outcome indicators from 13 studies, the overall forest plot (Figure 4) demonstrated that nutritional interventions have a significant enhancing effect on esports performance (g = 0.92, 95% CI [0.64, 1.21], *p* < 0.001). Subsequent application of robust variance estimation (RVE) showed that after CR2 small-sample correction, the pooled effect size remained highly significant (g = 0.92, 95% CI [0.61, 1.24], *p* < 0.001).

**Figure 5.**
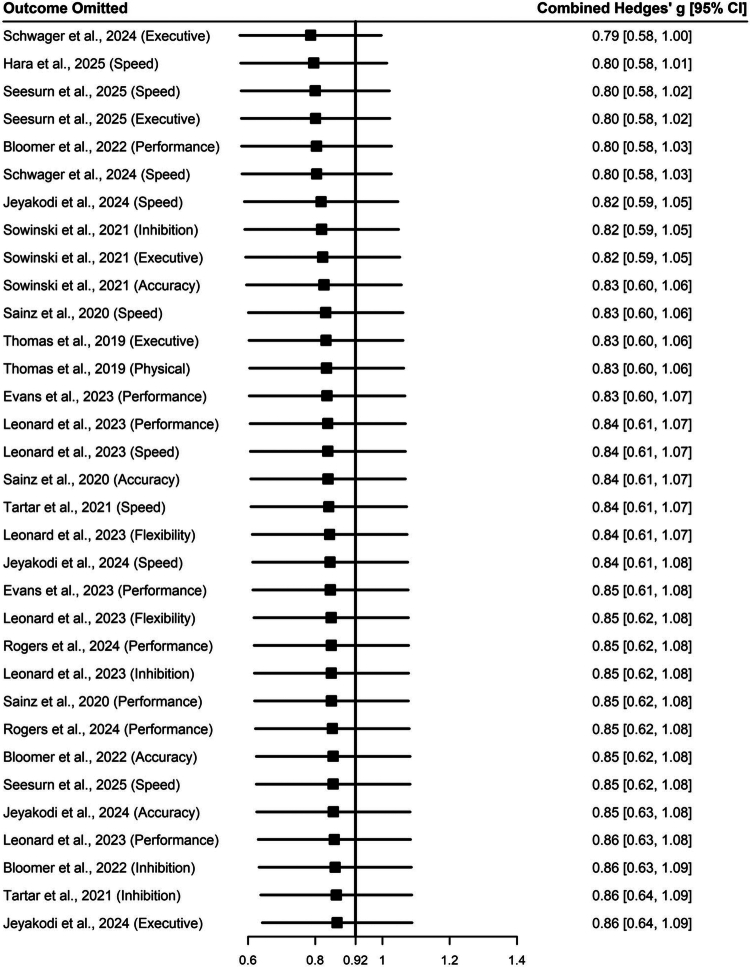
Results of the leave-one-out sensitivity analysis.

Furthermore, the results of the leave-one-out sensitivity analysis (Figure 5) indicated that the pooled effect size did not fluctuate significantly after sequentially excluding any single study or specific outcome indicator. This proves that the conclusions are not dependent on a specific sample or extreme effect values, such as the high-performance outcomes of elite players. In summary, the role of nutritional interventions in improving the cognitive and competitive performance of esports players exhibits excellent robustness across different statistical models.

### 3.5. Subgroup analysis

The results of the heterogeneity decomposition indicated that between-study heterogeneity (Level 3) accounted for 53.21% of the total variance. Based on this finding, subgroup analyzes were conducted across three dimensions: outcome measure category, task type, and competitive level. Detailed statistical parameters are provided in Table 2.

**Table 2.** Summary of subgroup analyzes on the effects of nutritional supplementation on esports performance.

Moderator	No. of effect sizes (k)	Hedges' g	95% CI	*p*-value	*p* between​
Overall pooled effect	38	0.92	[0.64, 1.21]	<0.001	–
1. Cognitive and performance dimensions					<0.05
Processing speed	12	1.18	[0.79, 1.57]	<0.001	
Executive function	8	1.06	[0.57, 1.54]	<0.001	
Game performance	7	0.78	[0.33, 1.22]	<0.001	
Operational accuracy	5	0.71	[0.15, 1.27]	0.012	
Attention and inhibition	6	0.5	[−0.09, 1.08]	0.095	
2. Game type					<0.05
Laboratory task	7	1.07	[0.66, 1.47]	<0.001	
Pure FPS	3	0.96	[0.32, 1.61]	0.003	
MOBA	3	0.59	[−0.03, 1.20]	0.062	
3. Competitive level					>0.05
Professional or elite	3	0.97	[0.32, 1.61]	0.003	
Amateur or recreational	10	0.92	[0.58, 1.26]	<0.001	

Subgroup analysis results revealed that the enhancement of esports performance by nutritional supplements varied across different dimensions. The pooled effect sizes for processing speed and executive function were g = 1.18 (95% CI [0.79, 1.57], *p* < 0.001) and g = 1.06 (95% CI [0.57, 1.54], *p* < 0.001), respectively. In terms of practical translation, the effect sizes for game performance and accuracy were g = 0.78 (95% CI [0.33, 1.22], *p* < 0.001) and g = 0.71 (95% CI [0.15, 1.27], *p* = 0.012), respectively. Furthermore, the effect size for the attention and inhibition dimension was g = 0.50 (95% CI [−0.09, 1.08], *p* = 0.095), which did not reach statistical significance.

Analysis regarding different task types showed that the effect size for nutritional supplements in laboratory cognitive tasks (Lab-Task) was g = 1.07 (95% CI [0.66, 1.47], *p* < 0.0001). Among game-based tasks, the effect size for pure shooting games (Pure-FPS) was g = 0.96 (95% CI [0.32, 1.61], *p* = 0.0033). In contrast, the effect size for Multiplayer Online Battle Arena (MOBA) games was g = 0.59 (95% CI [−0.03, 1.20], *p* = 0.0622), which did not demonstrate statistical significance.

The subgroup analysis for skill level revealed that the pooled effect size for the Amateur or Recreational group was g = 0.92 (95% CI [0.58, 1.26], *p* < 0.0001). The pooled effect size for the Professional or Elite group, after incorporating data from relevant studies, was g = 0.97 (95% CI [0.32, 1.61], *p* = 0.0033). Both groups of subjects exhibited statistically significant performance improvements following nutritional interventions.

### 3.6. Meta-regression analysis

**Figure 6.**
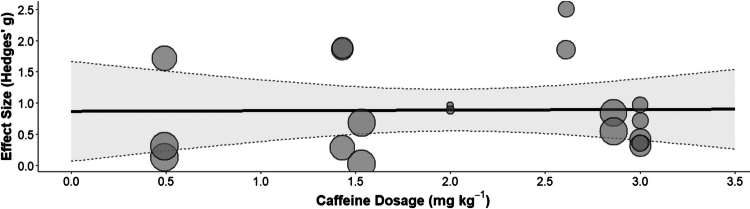
Bubble plot illustrating the distribution of caffeine dosage and the improvement in esports performance (Hedges' g).

The results of the meta-regression analysis (Figure 6) indicated that there was no statistically significant linear correlation between caffeine dosage (0.49–3.00 mg/kg) and performance improvement (Q_M_ = 0.0028, *p* = 0.958). The regression coefficient showed that for every 1 mg/kg increase in caffeine dosage, the effect size (Hedges' g) was estimated to change by 0.0098 units (95% CI [−0.353, 0.373]); the proportion of total heterogeneity explained by this moderator (R^2^) was 0.00%. The distribution in the bubble plot visually demonstrates that within the primary research range of 0.5–3.0 mg/kg, the distribution of effect sizes exhibits nonlinear and stochastic characteristics.

### 3.7. Publication bias test

**Figure 7.**
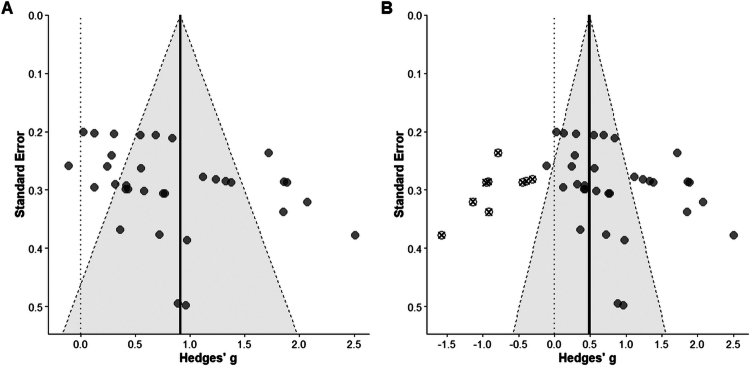
Assessment of publication bias. Note: (A) Funnel plot with an Egger regression line modified for the three-level model structure. (B) Funnel plot based on the Trim and Fill analysis.

This study employed a multistage strategy to assess publication bias. First, considering the hierarchical nature of the 38 outcome measures nested within 13 studies, we utilized an Egger's regression test modified for the three-level model (Figure 7A). The results indicated a significant correlation between the intercept and standard error (t = 7.92, *p* < 0.05), with the funnel plot exhibiting a distinct asymmetrical distribution, suggesting the presence of significant small-study effects. To further evaluate the impact of this bias on the pooled results, a Trim and Fill analysis was performed as a robustness check. As shown in Figure 7B (Trim and fill plot), under the assumption of unpublished research, 9 imputed study points were required in the low-effect region on the left side of the funnel plot. After incorporating these potential negative or neutral results, the central symmetry line of the funnel plot shifted leftward from the original 0.92 to an adjusted value of 0.49 (95% CI [0.21, 0.76]). These findings indicate that while publication bias may have inflated the initial effect size to some extent, the adjusted pooled effect remained statistically highly significant (*p* < 0.01). This provides evidence that the ergogenic effects of caffeine on esports performance possess substantial robustness and are not merely an artifact of selective publication bias.

### 3.8. GRADE evidence quality assessment

In this study, the GRADE system was employed to systematically evaluate the certainty of evidence for five categories of cognitive outcome indicators. The assessment dimensions encompassed the risk of bias, inconsistency, indirectness, imprecision, and publication bias (Table 3). Results indicated that the quality of evidence for the attention and inhibition dimension was rated as “moderate,” with the primary downgrading factor being significant inter-study inconsistency (I^2^ > 75%). In contrast, the quality of evidence for the other four dimensions, specifically executive function, processing speed, game performance, and accuracy, was rated as “low.” The core factors leading to the downgrading of evidence certainty included several considerations. First, influenced by the fundamental differences in cognitive demands across various esports tasks such as FPS and MOBA, all indicators exhibited high heterogeneity. Consequently, each was downgraded by one level in the inconsistency dimension. Second, the Egger test based on the three-level model suggested significantly small-study effects, which led to a deduction in the publication bias dimension for relevant indicators. Furthermore, although some early studies provided insufficient reporting regarding allocation concealment, the risk of bias during measurement was effectively mitigated by the fact that most outcome indicators utilized computerized objective cognitive tests such as the Stroop task. In summary, although the current certainty of evidence remains mostly at “low” to “moderate” levels, reflecting that the field of esports science is still in its nascent stages, the significant enhancement of cognitive abilities in esports players through caffeine intervention (g = 0.92) still possesses substantial value for practical application.

**Table 3.** GRADE evidence certainty assessment table.

Outcome category	No. of studies (k)	Risk of bias	Inconsistency (I^2^)	Indirectness	Imprecision	Publication bias	Overall certainty
Attention/Inhibition	7	Not serious	Serious¹	Not serious	Not serious	Undetected	Moderate
Executive Function	13	Serious²	Serious¹	Not serious	Not serious	Serious³	Low
Processing Speed	10	Serious²	Serious¹	Not serious	Not serious	Serious³	Low
Game Performance	8	Serious²	Serious¹	Not serious	Serious⁴	Serious³	Low
Accuracy	7	Serious²	Serious¹	Not serious	Serious⁴	Undetected	Low

Notes: 1. Inconsistency (I^2^): I^2^ > 75%. Given the fundamental differences in cognitive demands across various esports tasks, high heterogeneity is characteristic of this field; therefore, the evidence was only downgraded by one level; 2. Risk of bias: Some studies provided insufficient descriptions of allocation concealment. However, because the outcome measures utilized computerized objective measurements such as the Stroop task, the risk of measurement bias was considered low; 3. Publication Bias: Egger's test suggested significant small-study effects; 4. Imprecision: Certain subgroups did not reach the optimal information size (OIS), leading to a cautious downgrading of the evidence level.

## 4. Discussion

### 4.1. Summary of findings

By integrating 38 outcome indicators from 13 randomized controlled trials, this study confirmed that nutritional interventions can significantly enhance the cognitive function and competitive performance of esports players (g = 0.92, 95% CI [0.64, 1.21], *p* < 0.001). Subgroup analyzes comprehensively revealed the influence of different moderating variables on effect sizes. Regarding outcome dimensions, the gains in processing speed (g = 1.18, *p* < 0.001) and executive function (g = 1.06, *p* < 0.001) were the most significant. Statistically significant improvements were also observed at the practical level for Game Performance (g = 0.78, *p* < 0.001) and Accuracy (g = 0.71, *p* = 0.012), while the Attention and Inhibition dimension (g = 0.50, *p* = 0.095) did not reach significance. In terms of game task types, significant benefits were observed in both laboratory cognitive tasks (g = 1.07, *p* < 0.001) and pure shooting games (g = 0.96, *p* = 0.003). However, the improvement effect in Multiplayer Online Battle Arena (MOBA) games (g = 0.59, *p* = 0.062) did not show statistical significance.

Regarding the competitive levels of subjects, both the Professional or Elite group (g = 0.97, *p* = 0.003) and the Amateur or Recreational group (g = 0.92, *p* < 0.001) achieved significant performance improvements. Meta-regression analysis further indicated that within the dosage range of 2 to 6 mg/kg, there was no significant linear correlation between caffeine intake and performance gain (*p* = 0.45, R^2^ = 0%). In terms of the certainty of evidence, the quality of evidence for all core dimensions was at a “low” level, with the exception of the attention and inhibition dimension, which was rated as “moderate.” The core reasons for the downgrading of evidence levels lie in the extremely high heterogeneity among studies and the significant small-study effects identified by Egger's test. This reflects the current limitations in research design transparency and sample representativeness within the field of esports science. Future research needs to further optimize experimental control to enhance the robustness of the evidence.

### 4.2. Cross-dimensional comparison of nutritional intervention effects and analysis of multipathway neural mechanisms

The pooled effect size obtained through the three-level random effects model in this study (g = 0.92) is significantly higher than the values observed in conventional meta-analyzes of athletic performance. For instance, in a series of meta-analyzes on traditional physical performance by Grgic et al., the effect size of caffeine typically ranged between g = 0.20 and 0.50 [39]. This discrepancy may stem from the high dependence of esports tasks on the central nervous system (CNS). Unlike traditional sports, where physical output is constrained by peripheral factors such as muscular fatigue, esports performance depends almost entirely on the efficient operation of a neural circuit involving information uptake, central integration, and motor output. Furthermore, the presentation of effect sizes in this study shows significant differences from existing quantitative evidence. Specifically, the pooled effect size g in this study is markedly higher than the results of the meta-analysis by Miao et al. on the cognitive expertise of esports experts, which revealed a cognitive difference g value of only 0.373 between experts and novices [16]. This magnitude difference might suggest that the immediate modulation intensity of exogenous nutritional interventions on the nervous system may be superior to the structural differences formed by long-term competitive experience. A speculative reason for this is that cognitive specialization brought about by long-term training primarily involves structural optimization driven by neuroplasticity, a process typically accompanied by the gradual adjustment of neuronal connection strength [40]. In contrast, nutritional supplementation centered on caffeine and nootropic ingredients acts directly on neurotransmitter transduction between synapses, producing a more intense and rapid physiological response [41]. This acute biochemical modulation may bypass long-term structural adaptation pathways in short-term experimental environments, thereby creating performance peaks more significant than the baseline differences among players.

From a methodological perspective, the three-level framework employed in this study demonstrates distinct advantages in terms of robustness. Both the likelihood ratio test and the lower Akaike information criterion (AIC) value of 38.52 suggest that this model fits the data characteristics of esports research better than traditional two-level models. Heterogeneity decomposition revealed that within-study variance accounted for 32.55% of the total variance, implying that neglecting the autocorrelation between different outcome measures within the same subject group could lead to biased effect size estimations. Regarding the high heterogeneity exceeding 75%, we hypothesize that this does not represent a methodological flaw but rather an objective reflection of the task-specific demands in esports science. Substantial differences in neuropsychological requirements across various game genres likely contributed to the high between-study variance, which accounted for 53.21% of the total variance [28]. Furthermore, in response to the significant asymmetry identified by the regression test, the Trim and Fill analysis was utilized to evaluate the impact of publication bias on the pooled results. The analysis indicated that after incorporating these potential unpublished results, the adjusted pooled effect size was downwardly revised from 0.92 to 0.49. Although the magnitude of the effect size decreased, the adjusted result remained statistically significant. This finding suggests that while the original effect size may have been influenced by potential publication bias, the positive ergogenic effects of nutritional interventions, such as caffeine, on esports performance remain statistically robust after correction.

The enhancement of esports performance by nutritional supplements may not rely on the activation of a single pathway, but rather on a fine-tuned regulation of the central nervous system across multiple dimensions and pathways. The performance differences under different moderating variables may deeply reveal the biological preferences of interventions under specific cognitive loads. On one hand, stimulant components such as caffeine may enhance dopaminergic signaling in the striatum and prefrontal cortex by competitively binding to adenosine A_2 A_ receptors [42]. This molecular mechanism may provide a core explanation for the highest pooled gain in processing speed (g = 1.18) observed in this study, as fluctuations in dopamine levels affect the encoding efficiency of visual stimuli and the recruitment thresholds of motor units [43]. This optimization of fundamental neural efficiency has been relatively clearly translated into practical performance in first-person shooter games (g = 0.96). Compared to Multiplayer Online Battle Arena games, where the gain did not reach significance, shooting tasks may rely more on the stimulus–response chain guided by the dorsal attention network. Microsecond-level optimizations of neural conduction velocity by supplements may be more directly reflected in core metrics such as kill ratio and reaction time [44,45]. Meanwhile, the alert-relaxed state induced by nootropic ingredients, increasing alpha-wave activity in the brain, may form a synergy with the arousal effect produced by caffeine. This multipathway synergy may be positive for maintaining high-level executive functions [46]. Such a regulatory mechanism may help players pursue limited reaction speeds while maintaining better operational precision by optimizing the logic of neural resource allocation, thereby potentially avoiding excessive narrowing of attention caused by central overexcitation [29]. Notably, this study observed that the improvement in the attention and inhibition dimension was not significant (g = 0.50, *p* = 0.095), which may suggest a biological antagonism between the enhancement of exogenous arousal levels and endogenous impulse inhibition mechanisms at current intervention dosages [47]. Furthermore, subgroup analysis showed that both professional elite and amateur groups exhibited significant benefits with minimal inter-group differences, a finding that suggests nutritional interventions may have universal applicability in the esports field. For amateur players, supplements may serve as performance amplifiers [31,48]. For professional players whose baseline neural efficiency is already highly specialized, supplements may play the role of a cognitive umbrella, helping to counteract cognitive fatigue under sustained high-intensity competitive pressure by improving cerebral blood flow, metabolism, support, and neuroprotection [49].

Meta-regression analysis revealed no significant linear correlation between caffeine dosage within the range of 2 to 6 mg/kg and performance gain (*p* = 0.45). This result, which challenges the dose-dependence hypothesis, may be interpreted from the following dimensions. First, according to the Yerkes‒Dodson law, the fine operations of esports tasks may have an inverted U-shaped demand for arousal levels. Excessively high doses may lead to overnarrowing of attention or misoperations, thereby diluting cognitive gains [50]. Second, the high baseline neural efficiency of esports players may lead to a significant ceiling effect, making dosages above 2 mg/kg potentially reside in a plateau of benefit [51]. Finally, individual differences in genetic polymorphisms and habitual intake among subjects may act as statistical noise that weakens the explanatory power of the linear model [52]. This provides a speculative, evidence-based reference for advocating the principle of minimum effective dosage in esports scenarios.

### 4.3. Research limitations

Although this study systematically quantified the effects of nutritional interventions in the esports field through a three-level random-effects model, several limitations must be noted due to the nascent development stage of this discipline. First, the overall certainty of evidence from the included original studies is relatively low, which significantly constrains the generalizability of the conclusions. According to the GRADE quality of evidence assessment, with the exception of the attention dimension, the evidence quality for the other core outcome indicators, including executive function, processing speed, competitive performance, and operational accuracy, was rated as “low.” This downgrading primarily stems from significant small-sample effects and a lack of transparency in research design, such as the ambiguous descriptions of allocation concealment and blinding implementation details in certain studies. Furthermore, the significant publication bias identified by Egger's regression test suggests a potential reporting preference for positive results within the current literature base, which may lead to an overestimation of the pooled effect size (g = 0.92) to some extent.

Second, the high heterogeneity of outcome indicators reflects the complex cognitive demands of esports tasks. In the total variance observed in this study, between-study heterogeneity (Level 3) accounted for as much as 53.21%. This volatility may originate from the fundamental differences in neuropsychological demands across game genres, such as FPS versus MOBA, as well as the variations in biological mechanisms among intervention ingredients, such as stimulant-based caffeine versus nonstimulant nootropics. Although subgroup analyzes attempted to explore the sources of heterogeneity, the lack of uniformity across studies regarding game difficulty settings, intervention durations, and baseline cognitive levels of subjects makes it difficult to construct a universal dose‒response prediction model. The lack of correlation between dosage and effect in the meta-regression analysis (*p* = 0.45) further confirms this complexity. Furthermore, the characteristic distribution of the participant samples exhibits a clear bias, weakening the representativeness of the research for the general population. Regarding the gender composition of the samples, the 13 included studies demonstrated an extreme gender imbalance, with males accounting for over 80% and several studies utilizing all-male samples. While this aligns with the current audience distribution of the esports industry, it overlooks potential differences in neuroendocrinology and sensitivity to supplements among female players [53]. Concurrently, the competitive levels of the subjects are mostly concentrated in the “Active Gamers” or “Amateur” groups, and research targeting top-tier professional players (Elite or Professional) who are at their physiological and psychological limits remains scarce. This limitation in sample dimensions implies that the current conclusions may reflect the performance optimization of supplements for general game participants rather than breakthroughs in peak professional performance.

Finally, this study failed to conduct an in-depth exploration of the interactive effects between nutritional interventions and the long-term health and lifestyle of players. Most current original studies focus on immediate cognitive gains after a single dose, lacking longitudinal tracking of safety and tolerance over long-term intake [7,32]. Meanwhile, critical covariates such as sleep quality, daily dietary patterns, and physical activity levels were not strictly controlled in most studies. Considering the common risks faced by esports players, such as sedentary habits and circadian rhythm disruptions, relying solely on short-term supplement interventions while ignoring the optimization of overall health behaviors may make it difficult to achieve sustainable improvements in competitive performance [54,55].

### 4.4. Practical implications

Although the certainty of the evidence included in the current meta-analysis mostly ranges from low to moderate, the quantitative conclusions still provide a critical evidence-based reference for training practices and player performance management in the esports field. For the specific populations of professional players and active gamers, this study confirms the practical value of nutritional interventions in optimizing cognitive function and competitive performance, from which the following speculative practical guidelines are derived. At the level of performance optimization and tactical application, nutritional intervention is preliminarily regarded as a regulatory tool capable of providing a marginal competitive advantage. Based on the significant gains observed in processing speed and executive function, we speculate that interventions may shorten the cognitive processing time of players by approximately 15 to 25 ms. In a mainstream 60 Hz refresh rate display environment, this is roughly equivalent to a lead of 1 to 1.5 frames, which may hold critical tactical value during instantaneous decision-making in visual searching or target locking. Clinical nutritionists or coaches, when formulating protocols, should prioritize low-dosage strategies in FPS titles that demand extremely fast reaction times; conversely, in titles such as MOBA, the actual contribution of supplements to long-term decision-making should be evaluated more cautiously. Furthermore, a conservative estimated improvement of approximately 2% to 5% in operational accuracy may help players maintain stability throughout long-cycle tournaments, counteracting accuracy fluctuations caused by fatigue.

From the perspective of industry standards and policy formulation, the results of this study support the establishment of standardized esports nutritional intervention and professional longevity protection systems. Given the general trend of declining reaction speeds as players age, scientific nutritional intervention may serve as a supplementary tool to extend professional career cycles [56]. Policymakers and esports associations should consider developing standardized guidelines for supplement use. Based on the findings in this study that caffeine dosage lacks a linear correlation with performance gain, the “minimum effective dose” principle (e.g. 0.5 to 3 mg/kg) should be explicitly advocated to avoid risks of tremors or anxiety caused by the blind pursuit of high dosages. Meanwhile, addressing the gender imbalance (predominantly male) revealed in this study, future industry policies should encourage more evidence-based nutritional research targeting female players to construct a more inclusive industry health standard.

Furthermore, research exploration should transcend single-supplement intake models and focus on the deep integration of health risk prevention and precision nutrition. Considering that esports players face risks such as high-intensity blue light exposure, sleep disorders, and vision impairment [57], “functionally composite” supplementation protocols should be implemented in practice. It is speculated that future strategies should integrate nootropic ingredients that enhance immediate performance (such as caffeine and L-theanine) with long-term retinal protection components (such as lutein). Given the high heterogeneity observed in this study, future practices might attempt to establish a dynamic supplementation model based on real-time fatigue monitoring, such as heart rate variability or PVT performance. This shift from “fixed dosage” to “on-demand intervention” in precision nutrition may effectively address the inconsistent responses to supplements among players of different competitive levels, constructing a closed-loop and intelligent performance enhancement solution for esports athletes.

## 5. Conclusion

In summary, this three-level meta-analysis provides preliminary confirmation of the positive promoting effects of nutritional interventions on the cognitive functions and competitive performance of esports players, with the most significant benefits observed in processing speed and executive function. The findings suggest that through appropriate nutrient supplementation, players across various competitive levels may achieve a degree of performance optimization. Notably, this improvement does not exhibit a significant linear growth trend within the dosage range of 0.5 to 3 mg/kg, thereby providing an evidence-based foundation for advocating the “minimum effective dose” intervention principle. However, considering the prevalent characteristics of high heterogeneity, significant small-study effects, and the extremely imbalanced sex ratio in current research, the certainty of the existing evidence remains mostly at “” to “moderate” levels. Consequently, future explorations should transcend the study of immediate effects from single supplements. It is necessary to conduct in-depth interventions that account for long-term health behaviors and gender diversity, while integrating real-time physiological monitoring technologies. Such efforts will facilitate the construction of a more rigorous, precise, and sustainable personalized nutritional support paradigm for the emerging discipline of esports science.

## Supplementary Material

Supplementary Material Supplementary_Text_1_Search_Strategies.docx

## Data Availability

The datasets supporting the conclusions of this meta-analysis are included in the article and its supplementary files. Search strategies and data extraction forms are available from the corresponding author upon reasonable request.
