# Supplementary material for: Effects of nutritional interventions on competitive performance and dose‒response relationships among esports players of different skill levels: a systematic review and three-level meta-analysis
Source: J Int Soc Sports Nutr. 2026 Apr 24;23(1):2663363. doi: 10.1080/15502783.2026.2663363 (PMC13112869; doi:10.1080/15502783.2026.2663363)
Supplement: Supplementary Material — Supplementary_Text_1_Search_Strategies.docx [file RSSN_A_2663363_SM7374.docx]

**Supplementary Text 1 Search Strategies**

### **1.PubMed**

**search strategy：**

#1 ("Video Games"[Mesh] OR "Esports"[tiab] OR "E-sports"[tiab] OR "Gamer*"[tiab] OR "video gaming"[tiab] OR "video game*"[tiab])

#2 ("Dietary Supplements"[Mesh] OR "Caffeine"[Mesh] OR "Nutrition"[tiab] OR "Supplement*"[tiab] OR "Placebo"[tiab] OR "Caffeine"[tiab] OR "Drink*"[tiab] OR "Capsule*"[tiab] OR "Nootropic*"[tiab])

#3("Randomized Controlled Trial"[pt] OR "Randomized"[tiab] OR "Controlled"[tiab] OR "Crossover"[tiab] OR "Random allocation"[tiab])

#4 #1 AND #2 AND #3

### **2.Web of Science**

### **search strategy：**

#1 TS=("Video Game*" OR "Esports" OR "E-sports" OR "Gamer*" OR "video gaming")

#2 TS=("Dietary Supplement*" OR "Caffeine" OR "Nutrition" OR "Supplementation" OR "Placebo" OR "Drink*" OR "Capsule*" OR "Nootropic*")

#3 TS=("Randomized Controlled Trial" OR "Randomized" OR "Controlled" OR "Crossover" OR "Random allocation")

#4 #1 AND #2 AND #3

### **3.Embase**

### **search strategy：**

#1 ('video game'/exp OR 'esports':ti,ab OR 'e-sports':ti,ab OR 'gamer*':ti,ab OR 'video gaming':ti,ab OR 'video game*':ti,ab)

#2 ('dietary supplement'/exp OR 'caffeine'/exp OR 'nutrition':ti,ab OR 'supplement*':ti,ab OR 'placebo':ti,ab OR 'drink*':ti,ab OR 'capsule*':ti,ab OR 'nootropic agent'/exp)

#3 ('randomized controlled trial'/exp OR 'randomization'/exp OR 'randomized':ti,ab OR 'controlled':ti,ab OR 'crossover procedure'/exp OR 'crossover':ti,ab)

#4 #1 AND #2 AND #3

### **4.Cochrane Library**

### **search strategy：**

#1 [mh "Video Games"] OR (Esports):ti,ab,kw OR (E-sports):ti,ab,kw OR (Gamer*):ti,ab,kw OR (video gaming):ti,ab,kw OR (video game*):ti,ab,kw

#2 [mh "Dietary Supplements"] OR [mh "Caffeine"] OR (Nutrition):ti,ab,kw OR (Supplement*):ti,ab,kw OR (Placebo):ti,ab,kw OR (Drink*):ti,ab,kw OR (Capsule*):ti,ab,kw OR (Nootropic*):ti,ab,kw

#3 [mh "Randomized Controlled Trials as Topic"] OR (randomized):ti,ab,kw OR (controlled):ti,ab,kw OR (Crossover):ti,ab,kw

#4 #1 AND #2 AND #3

### **5.Scopus**

### **search strategy：**

#1 TITLE-ABS-KEY ("Video Game*" OR "Esports" OR "E-sports" OR "Gamer*" OR "video gaming")

#2 TITLE-ABS-KEY ("Dietary Supplement*" OR "Caffeine" OR "Nutrition" OR "Supplementation" OR "Placebo" OR "Drink*" OR "Capsule*" OR "Nootropic*")

#3 TITLE-ABS-KEY ("Randomized Controlled Trial" OR "Randomized" OR "Controlled" OR "Crossover" OR "Random allocation")

#4 #1 AND #2 AND #3

### **6.CINAHL**

### **search strategy：**

#1 ( (MH "Video Games") OR TI ( Esports OR "E-sports" OR Gamer* OR "video gaming" OR "video game*" ) OR AB ( Esports OR "E-sports" OR Gamer* OR "video gaming" OR "video game*" ) )

#2 ( (MH "Dietary Supplements") OR (MH "Caffeine") OR TI ( Nutrition OR Supplement* OR Placebo OR Drink* OR Capsule* OR Nootropic* ) OR AB ( Nutrition OR Supplement* OR Placebo OR Drink* OR Capsule* OR Nootropic* ) )

#3 ( (MH "Randomized Controlled Trials") OR TI ( Randomized OR Controlled OR Crossover OR "Random allocation" ) OR AB ( Randomized OR Controlled OR Crossover OR "Random allocation" ) )

#4 #1 AND #2 AND #3
